# Auswirkungen der Corona-Krise auf die europäische Klimaschutzpolitik

**DOI:** 10.1007/s10273-020-2656-9

**Published:** 2020-05-20

**Authors:** Thomas M. Treptow

**Affiliations:** BWL, iba - Duales Studium Nürnberg, Zeltnerstraße 19, 90443 Nürnberg, Deutschland

**Keywords:** E61, Q54, E22, Q52

## Abstract

Neben vielen anderen negativen Folgen hat die Corona-Krise auch direkte Auswirkungen auf den EU-weiten Handel mit Emissionszertifikaten für Treibhausgasemissionen. Dieser stellt einen Eckpfeiler der europäischen Klimaschutzpolitik dar. Den kurzfristig geringer zu erwartenden Treibhausgasemissionen stehen aufgrund der erwarteten Rezession mittel- und langfristig niedrigere Preise für Emissionsrechte gegenüber. Diese machen die gegenwärtigen Produktionstechnologien tendenziell attraktiver. Dadurch wird eine wesentliche Zielsetzung europäischer Klimaschutzpolitik, der ökonomisch-rationale Wechsel der Unternehmen auf klimafreundlichere Produktionstechnologien, erschwert.

## Abstract (en)

The actual Corona crisis has a negative impact on the economic situation of the affected economies. This has direct consequences for the EU-wide trading of greenhouse gas emission allowances, which is an important building block of European climate change policy. In contrast, although there is an expected short-term decrease in the volume of greenhouse gas emissions due to the emerging economic recession, we should also expect lower prices of emission allowances in the mid to long term, which will make current production technologies more attractive. One important goal of European climate change policy — changing existing manufacturing technologies to less greenhouse gas emitting alternatives — will become even more difficult.

Die Auswirkungen der Corona-Krise sind kaum zu überschätzen. Neben den tragischen Folgen für Gesundheit und Leben der Weltbevölkerung werden sich weitere Auswirkungen ergeben. Diese betreffen die Wirtschaft aber auch die Ökologie. Der sich ausbreitende Coronavirus und die daraus resultierenden Infektionen der Menschen sowie der wirtschaftliche Shutdown sind dafür verantwortlich, dass sich eine Vielzahl der Volkswirtschaften auf dem Weg in die Rezession befindet. Durch den temporären Angebotsschock wird das volkswirtschaftliche Produktionsniveau in der nächsten Zukunft deutlich niedriger als prognostiziert ausfallen. Die Investitionsnachfrage der Unternehmen wird durch die Unsicherheit über die zukünftige ökonomische Entwicklung weiter belastet. Je länger die Krise dauert, umso mehr ist zu erwarten, dass auch die privaten Haushalte einen nachhaltig negativen Einfluss auf ihre Einkommenssituation erfahren werden. Damit wird ebenfalls die private Konsumnachfrage zurückgehen. Aus diesen nachteiligen Auswirkungen auf die Ökonomien in Europa ergeben sich auch nachteilige Einflüsse auf die europäische Klimaschutzpolitik, und die Realisierung der zukünftigen Klimaschutzziele wird deutlich erschwert.

**Dr. Thomas M. Treptow** ist Dozent an der Internationalen Berufsakademie, iba – University of cooperative education in Nürnberg.

## Das Emissionshandelssystem der EU

Ein zentrales Instrument der europäischen Klimaschutzpolitik ist der Emissionshandel, das sogenannte Emissions Trading System (ETS). 2005 hat die Europäische Union Emissionsberechtigungen für die Emission von Treibhausgasen definiert und eingeführt. Danach müssen treibhausgasemittierende Unternehmen, wie Kraftwerksbetreiber, produzierende Unternehmen und seit 2012 auch Fluggesellschaften in den EU-Mitgliedstaaten, Norwegen, Island und Liechtenstein pro emittierter Tonne Treibhausgase ein Emissionszertifikat als Berechtigung nachweisen bzw. einlösen.

Durch die sukzessive Reduzierung der Gesamtzahl vorhandener Emissionsrechte um jährlich 1,74 % bis 2020 und ab 2021 um 2,2 % und die Möglichkeit, diese Emissionsrechte zu handeln, erhalten die betroffenen Unternehmen einen ökonomischen Anreiz, ihre Treibhausgasemissionen kostenminimal zu steuern. Idealtypisch bestehen für die Unternehmen dabei die folgenden, kombinierbaren Möglichkeiten:

- Einsatz verbesserter Produktionstechnologien, die zu weniger Emissionen führen,
- Einschränkung emissionsverursachender Produktion,
- Erwerb von Emissionszertifikaten für unveränderte Produktionstechnologien.

Aus ordnungspolitischer Sicht handelt es sich beim ETS um ein marktwirtschaftliches Instrument der Umweltpolitik, das für ein festgelegtes zulässiges Emissionsvolumen von Treibhausgasen für die verpflichteten Unternehmen eine marktseitige Preisfindung für die gehandelten Emissionszertifikate erlaubt. Durch die sukzessive Absenkung der zulässigen Emissionsmengen werden die zugrundeliegenden Klimaschutzziele durch diesen sogenannten „cap & trade“-Ansatz verfolgt und potenziell erreicht. Für ein festgelegtes Emissionsvolumen pro Periode führt dies unter bestimmten theoretischen Annahmen zu einem Marktpreis der Emissionszertifikate, der dem optimalen Steuersatz einer entsprechenden Treibhausgassteuer, der Pigou-Steuer, entspricht.

Dem ETS wird regelmäßig und aktuell auch noch einmal durch den bekanntgegebenen European Green Deal der Europäischen Kommission eine hohe Bedeutung bei der Erreichung der Klimaschutzziele zugeschrieben. Bereits in der Vergangenheit haben sich jedoch aufgrund konjunktureller Entwicklungen Herausforderungen für die Verfolgung der klimapolitischen Ziele durch das ETS gezeigt.

Bereits in der dritten, aktuell noch laufenden Handelsperiode des ETS (2013 bis 2020) wurden 900 Mio. Emissionsrechte des seit 2009 aufgebauten Überschussangebots zunächst in die Subperiode 2019 bis 2020 verschoben und dann ab 2019 der neu gegründeten Marktstabilisierungsreserve zugeordnet. Damit sollte vermieden werden, dass dieser nicht nachgefragte Angebotsteil den Preis der Emissionszertifikate drückt. Die Marktstabilisierungsreserve stellt ein Fondsvehikel dar, um die gewünschten Preissignale des ETS für Treibhausgasemissionen zu fördern. Eine vergleichbare und sehr wahrscheinlich quantitativ bedeutsamere Situation ergibt sich jetzt als Auswirkung der durch die Corona-Krise verursachten Rezession.

## Auswirkungen der Corona-Krise auf den Preis von Emissionszertifikaten

Die Summe der benötigten Emissionsrechte stellt für ein Unternehmen bei gegebener Produktionstechnologie einen zwingend einzusetzenden Produktionsfaktor dar, der Kosten verursacht. Prognostizieren die Unternehmen aufgrund des verschlechterten ökonomischen Ausblicks ein geringeres Produktionsniveau, so benötigen sie auch weniger Emissionszertifikate bzw. sind die schon vorhandenen Emissionszertifikate für einen längeren Planungshorizont ausreichend. (Die Auswirkungen auf den ETS-Teilmarkt der Emissionsberechtigungen für europäische Fluggesellschaften sind aufgrund der stark eingeschränkten Flugpläne ebenfalls als hoch zu erwarten.)

Dies führt isoliert betrachtet zunächst dazu, dass die Nachfrage nach Emissionszertifikaten sinkt. Der kurzfristige Vorteil für die Realisierung der Klimaschutzziele besteht also darin, dass durch eine geringere Produktion weniger Emissionen verursacht werden.

Gleichzeitig entstehen im ETS jedoch Effekte mit mittel- bis langfristiger Wirkung. Für ein im Regelbetrieb unelastisches Angebot von Emissionszertifikaten bedeutet eine geringere Nachfrage fallende Preise. Langfristig planende Unternehmen, die unter das ETS fallen, berechnen für sinkende Kosten von Emissionszertifikaten einen höheren Kapitalwert ihrer gegenwärtigen Produktionstechnologie. Es ergibt sich damit ein Steuerungsimpuls, der die gegenwärtige treibhausgasemittierende Produktionsweise tendenziell vorteilhafter gegenüber treibhausgasvermeidenden Alternativen macht. Ist dieser Vorteil auch absolut gegeben, ist zu erwarten, dass diese Unternehmen aktuell Emissionszertifikate für ihre zukünftigen Emissionen bei unveränderter Produktionstechnologie günstig erwerben oder sich den späteren Erwerb über Derivate, beispielsweise Future-long-Transaktionen, zum aktuellen Preis sichern.

Dass solche investiven Kalküle aktuell umgesetzt werden, lässt sich beispielsweise für die Transaktionsvolumina und die Preisentwicklung der Emissionszertifikate an der European Energy Exchange, Leipzig, eine der Börsen, an denen der Handel von Emissionszertifikaten stattfindet, im März unterstellen. In der Zeit vom 10. bis 17. März 2020 war im Spotmarkt und auf Tagesbasis ein Anstieg des Transaktionsvolumens von 29.000 auf 370.000 Emissionszertifikate bei einem fallenden Transaktionspreis von ca. 24 Euro auf 18,24 Euro pro Emissionszertifikat beobachtbar (vgl. Abbildung 1).

Ordnungspolitisch ist dies für das ETS als marktwirtschaftliches Instrument und einer darauf basierenden Regulierung der betroffenen Unternehmen nicht zu kritisieren. Ebenfalls darf dabei nicht vergessen werden, dass die Unternehmen bei ihren bereits im Bestand befindlichen Emissionszertifikaten durch den Preisverfall einen massiven Rückgang der Marktwerte bei gleichzeitig gestiegener Unsicherheit über den zukünftigen Verwendungszeitpunkt zu verzeichnen haben. In den angesprochenen sieben Tagen hat allein auf Grundlage der obigen Daten ein Preisrückgang von 24 % bei wahrscheinlich gleichzeitig zugenommener Preisvolatilität stattgefunden.

## Folgen für die Erreichung der Klimaschutzziele

Für die Erreichung der Klimaschutzziele ist das schnell gesunkene Preisniveau in vielfacher Hinsicht nachteilig, und die rezessiv bedingten Emissionseinsparungen sind damit teuer erkauft.

Abbildung 1
**Preisentwicklung und Transaktionsvolumina von Emissionsberechtigungen**

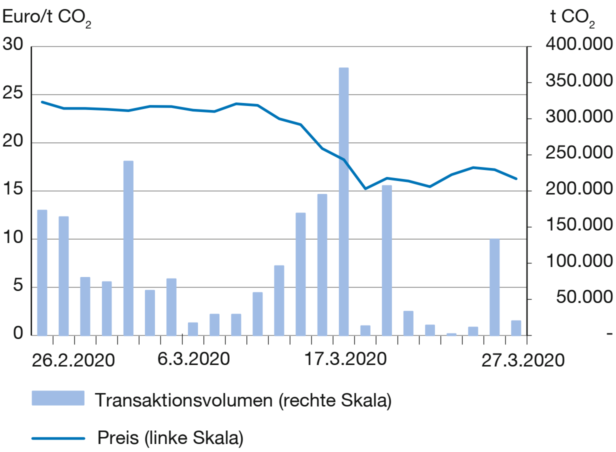

Quelle: EEX Group.

- Die unter das ETS fallenden Unternehmen sind tendenziell für einen längeren Zeitraum mit Emissionsberechtigungen ausgestattet. Somit wird eine den Preis treibende Nachfrage tendenziell länger nicht zu beobachten sein.

- Die für die Erreichung der Klimaschutzziele gewünschte Umstellung der Produktionstechnologien der Unternehmen wird aufgrund der gesunkenen Preise für Emissionszertifikate relativ teurer gegenüber der Beibehaltung etablierter Technologien. Damit wird der klimapolitisch gewünschte Wechsel der Produktionstechnologien erschwert.

- Die durch die gesamtkonjunkturelle Entwicklung erhöhte Unsicherheit, die bisher schon die Investitionsnachfrage der Unternehmen trotz niedrigster Zinsen beeinträchtigt hat, wird durch den Corona-Schock noch weiter verstärkt und führt zu einer zusätzlichen Absenkung der Kapitalwerte auch klimafreundlicher Investitionsprojekte.

Die denkbare Einschränkung des Angebots von Emissionszertifikaten für die nächste Handelsperiode bzw. eine Dotierung der Marktstabilisierungsreserve über die jährliche Reduktionsrate verfügbarer Emissionszertifikate hinaus, erhöht die Unsicherheit für die Unternehmen weiter. Grundsätzlich unterliegen insbesondere diskretionäre Politikmaßnahmen dabei der Gefahr, das Preissignal des ETS weiter und in nicht beabsichtigter Weise zu stören. Dies führt ebenfalls zu einer steigenden Investitionsunsicherheit bei den Unternehmen.

## Rückschlag für treibhausgasvermeidende Technologien

Zusammengefasst werden damit aufgrund des rezessionsbedingt geringen Produktionsniveaus kurzfristig deutlich geringere Treibhausgasemissionen die Folge sein als unter dem „cap & trade"-Ansatz des ETS bisher erwartet wurden. Allerdings erfahren die klimapolitisch beabsichtigten Substitutionseffekte gegenwärtiger Produktionsverfahren hin zu treibhausgasvermeidenden Produktionstechnologien einen Rückschlag. Die Regulierung der Treibhausgasemissionen in der EU durch ein marktwirtschaftliches Instrument wie das ETS ist weiterhin zu bejahen. Die komplexen Wechselwirkungen zwischen Politikeingriffen in das ETS und dessen Preissignalen für die Investitionsentscheidungen der Unternehmen verdeutlichen die dabei bestehenden wirtschafts- und klimapolitischen Herausforderungen. Dies gilt auch für die Ankündigung der EU-Kommission unter dem European Green Deal, die Reduzierungsziele der Treibhausgasemissionen für das ETS in verantwortungsvoller Weise zu erhöhen. Darin steckt allerdings auch die Chance ökologisch-ökonomisch vereinbarer Politikmaßnahmen.
